# DUAL ELIGIBILITY AND INJURY-RELATED EMERGENCY DEPARTMENT VISITS AMONG ASSISTED LIVING RESIDENTS

**DOI:** 10.1093/geroni/igac059.1655

**Published:** 2022-12-20

**Authors:** Cassandra Hua, Portia Cornell, Elizabeth White, Katherine Kennedy, Ian Nelson, Kali Thomas

**Affiliations:** Brown University School of Public Health, Providence, Rhode Island, United States; Providence VA Medical Center / Brown University, Providence, Rhode Island, United States; Brown University, Providence, Rhode Island, United States; Providence VA Medical Center, Providence, Rhode Island, United States; Miami University, Oxford, Ohio, United States; Brown University, Providence, Rhode Island, United States

## Abstract

Using 2018 Medicare data, we examined the relationship between dual eligibility and injury-related emergency department use among a cohort of assisted living residents (n=116,754). We fit multilevel models with random intercepts at the assisted living community and license type levels. The baseline rate of injury-related emergency department emergency department use was 0.17. After controlling for resident characteristics (i.e., age, sex, race, and chronic conditions), license type characteristics (i.e., dementia care licensure, staffing regulations), and assisted living community characteristics (i.e., size and percentage of residents with dementia), being dually eligible for Medicare and Medicaid was associated with a 12% increase in the probability of having an injury-related emergency department visit (b=.02; p<.001). Assisted living communities that serve duals may have fewer resources and staff to provide personal care, potentially leading to increased rates of injuries.

